# Functional genomics identifies novel genes essential for clear cell renal cell carcinoma tumor cell proliferation and migration

**DOI:** 10.18632/oncotarget.2097

**Published:** 2014-06-12

**Authors:** Christina A. von Roemeling, Laura A. Marlow, Derek C. Radisky, Austin Rohl, Hege E. Larsen, Johnny Wei, Heather Sasinowska, Heng Zhu, Richard Drake, Maciek Sasinowski, Han W. Tun, John A. Copland

**Affiliations:** ^1^ Department of Cancer Biology, Mayo Clinic Comprehensive Cancer Center, Jacksonville, Florida; ^2^ Division of Hematology & Oncology, Mayo Clinic Comprehensive Cancer Center, Jacksonville, Florida; ^3^ Incogen, Inc., Jacksonville, Florida; ^4^ MUSC Proteomics Center, Jacksonville, Florida; ^5^ Department of Pharmacology & Molecular Sciences, Johns Hopkins School of Medicine, Jacksonville, Florida

**Keywords:** Renal cell carcinoma, gene array, metastasis, therapeutic targets

## Abstract

Currently there is a lack of targeted therapies that lead to long-term attenuation or regression of disease in patients with advanced clear cell renal cell carcinoma (ccRCC). Our group has implemented a high-throughput genetic analysis coupled with a high-throughput proliferative screen in order to investigate the genetic contributions of a large cohort of overexpressed genes at the functional level in an effort to better understand factors involved in tumor initiation and progression.

Patient gene array analysis identified transcripts that are consistently elevated in patient ccRCC as compared to matched normal renal tissues. This was followed by a high-throughput lentivirus screen, independently targeting 195 overexpressed transcripts identified in the gene array in four ccRCC cell lines. This revealed 31 ‘hits’ that contribute to ccRCC cell proliferation.

Many of the hits identified are not only presented in the context of ccRCC for the first time, but several have not been previously linked to cancer. We further characterize the function of a group of hits in tumor cell invasion. Taken together these findings reveal pathways that may be critical in ccRCC tumorigenicity, and identifies novel candidate factors that could serve as targets for therapeutic intervention or diagnostic/prognostic biomarkers for patients with advanced ccRCC.

## INTRODUCTION

Renal Cell Carcinoma (RCC) is among the top ten most common solid tumors in the United States. In 2013 it was projected that over 60,000 new cases would be diagnosed, and that 13,000 deaths would result from this disease [1]. The clear cell subtype of RCC (ccRCC) manifests in the majority of all cases, accounting for approximately 80% [2]. The prognosis for patients who present with localized disease is relatively good, and treatment typically involves surgical resection of the tumor. Patients who present with advanced and metastatic disease have markedly worse prognoses, with overall survival rates dropping to 50% for stage III and <10% for stage IV [3]. This is attributed to disease recurrence as well as the drug and radiation resistant nature of ccRCC.

Unfortunately, ccRCC tends to be asymptomatic, and it is estimated that 20-30% of patients present with metastatic disease during initial diagnosis [4]. Furthermore, 20-30% of patients who undergo partial or whole nephrectomy as treatment for localized disease demonstrate recurrence of metastatic disease within 5 years of the original diagnosis [5]. This is thought to be the result of the highly invasive character of ccRCC, and that undetectable micro-metastases can even develop during early stages of disease [5]. In addition, ccRCC is highly resistant to radiation, chemotherapy, and targeted therapy [6, 7]. To date, there are several treatment courses that have been approved by the FDA; still, none are curative with the exception of patients who respond well to immunotherapy, which has demonstrated success in 5-6% of patients [7]. Drug resistance to targeted therapy also occurs rapidly. Treatment therefore is administered long-term, often in combination with other agents or applied sequentially, and requires management of toxicity [3, 7].

Currently, there remains a need for the development of new courses of targeted therapy. New tumor-specific properties that contribute to disease progression and can be exploited through pharmacologic intervention must be identified. Explorations into the genetic signatures of heritable and sporadic ccRCC have elucidated several genes thought to play a role in the initiation and progression of this disease, yet many of these remain to be functionally validated. Of those currently known, mutation or loss of the tumor suppressor Von Hippel Lindau (*VHL*) and resulting stabilization of the hypoxia inducible factor proteins (HIF) remains the best characterized, and manifests in 60-80% of all cases of ccRCC [8]. HIF stabilization is most commonly known to promote tumor angiogenesis via transcriptional upregulation of vascular endothelial growth factor (VEGF), platelet derived growth factor (PDGF), as well as several other genes [8]. Several of the approved therapies for ccRCC are agents that target these pro-angiogenic factors; however, tumor responses to these courses of therapy are short-lived. Other commonly investigated factors such as oncogenic *KRAS* or mutations in *TP53* rarely contribute to ccRCC [8, 9].

Our group has employed a high-throughput gene microarray screen to identify genetic transcripts that are over-expressed at all stages of ccRCC as compared to matched normal kidney tissue. A high-throughput lentiviral array was designed to individually target 195 of the most consistently over-expressed genes identified in the gene array in four established ccRCC cell lines. Measuring decreased proliferative capacity as a read-out for the lentiviral screen, we have identified 31 genes that are required for ccRCC cell propagation, many of which are unique. While little is currently known about the protein function of several of these gene products, many are implicated in metabolism, angiogenesis, differentiation, and cell motility in other cancer systems. Of these, we further establish a role for CDH13 in tumor angiogenesis, as well as a pro-migratory role for four novel factors including KISS1R, KSR1, CAMK1, and SSPN in ccRCC.

## RESULTS

### Comparative marker selection of gene array data reveals cohort of genes consistently over-expressed and down-regulated in ccRCC

**Figure 1:**
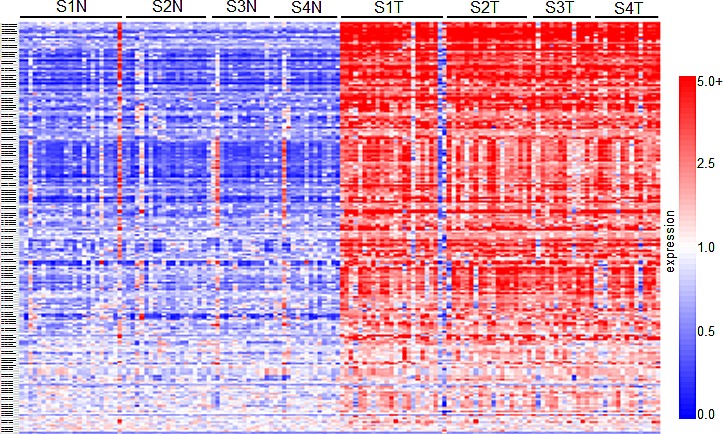
Results of a high-throughput gene-array screen evaluating gene transcript expression between ccRCC and normal matched patient tissue samples The heatmap summarizes the results of the comparative marker selection, and comprises 195 genes that demonstrate over-expression in tumor samples at all stages of disease. In total, 144 tissue samples were analyzed: 24 matched pairs from stage I, 19 from stage II, 14 from stage III, and 15 from stage IV. Rows are organized numerically by Affymetrix gene IDs, and columns are organized as stage I-IV normal (e.g. S1N, S2N) followed by tumor samples from left to right.

Comparative marker selection of the results of a high-throughput DNA microarray screen evaluating expression between matched normal renal tissue and ccRCC samples derived from stage I through IV patients was used to identify gene transcripts that are upregulated in diseased tissues (GSE-53757). This analysis revealed a total of 2,875 genes that are over-expressed (n≥2 fold change induction where p≤0.05), and 3,062 genes that are downregulated (n≤0.5 fold change decrease, where p≤0.05) in tumor samples when compared to matched normal (GSE-53757). Of these, 195 genes of interest that consistently demonstrated elevated expression as compared to normal levels were selected for further functional analysis. These selected genes are summarized in the heatmap in Figure 1. A list sorting the top 195 genes alphabetically is also provided (SF1).

### High-throughput targeted lentiviral screen of genes over-expressed in ccRCC reveals a subset that considerably affect tumor cell proliferation

**Figure 2:**
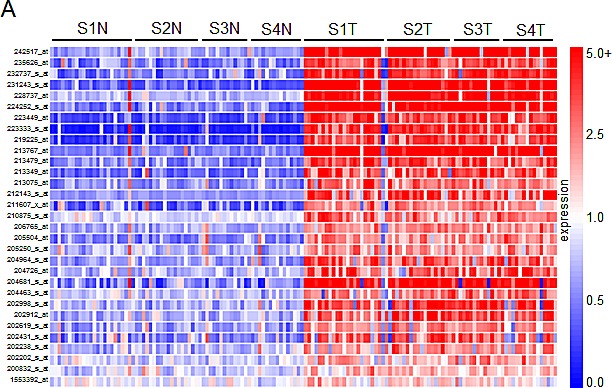

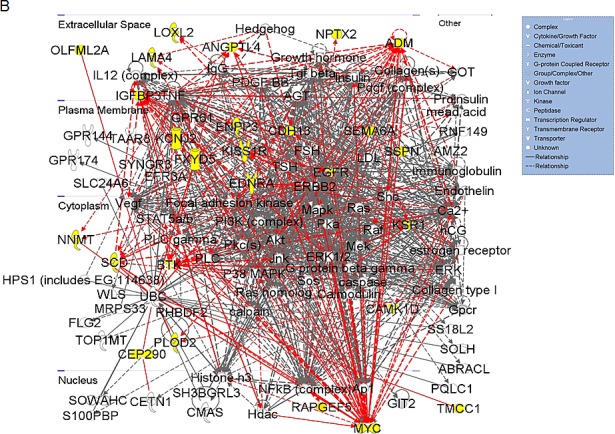
Gene network clustering of top hits (A) Heatmap of the 31 gene hits identified from the high-throughput lentiviral screen. In total, 144 tissue samples were analyzed: 24 matched pairs from stage I, 19 from stage II, 14 from stage III, and 15 from stage IV. Rows are organized numerically by Affymetrix gene IDs, and columns are organized as stage I-IV normal followed by tumor samples from left to right. (B) 26 of the 31 hits identified were sorted into one primary signaling network. Gene hits are highlighted in yellow. This network is organized by cellular compartment, and molecules which are known to interact directly are connected by a solid line, while indirect relationships are shown with a dashed line. Immediate up/downstream effectors of gene hits are connected by red lines. The gene products are further defined by functional molecules (see key).

We next sought to characterize which of the top 195 genes identified in the gene array screen contributed to tumor cell proliferation. A high-throughput lentiviral screen designed to target each individually was completed using MISSION® shRNA lentiviral particles from Sigma, arranged in 96-well format. *CCND1* (cyclin D1) and *MYC* oncogenes, both identified as overexpressed in the comparative analysis, were considered as positive controls due to their well characterized roles in cell proliferation [10]. Four established ccRCC cell lines including ACHN and Caki1 (*VHL* wt) and Caki2 and KIJ265T (*VHL* mut) were used in the lentiviral screen. Cyquant® fluorescence based assays were used to measure cell proliferation based upon DNA fluorescence. Additional details of the lentiviral screen and Cyquant® assays are further described in the methodology section. Hit selection was performed using a B-score (analog of Z score) statistical approach to minimize column and row bias using HTS corrector [11] (SF2). From these analyses, 31 gene ‘hits’ were identified whose targeted mRNA silencing yielded significant growth inhibition with 90-95% probability under normal distribution in at least three of the four cell lines examined. Hits are shown in alphabetical order along with Sigma clone references, with average B-score summaries (n=3) for each cell line provided (Table 1). The numeric values denoted under each cell line represent the polished standard deviation on a normal distribution scale. Each integer represents one standard deviation, with negative values corresponding to a decrease in proliferation. Values less than −1 represent a loss of more than 85% in total population when compared to control values. A heatmap depicting gene expression in normal samples as compared to tumor corresponding to all stages of disease for the 31 hits is shown (Figure 2A). Differential gene expression of the 31 hits was further validated in two other publically available, independent gene array datasets comparing ccRCC tissue and normal samples. Overexpression was observed in 28/31[12] of the gene hits in the first dataset, and 29/31[13] in the second (SF3-4).

**Table 1:** Average B score summary of gene hits Summary of the 31 gene hits identified to significantly decrease tumor cell proliferation. Clone references for the specific lentiviral constructs used to generate growth effects are shown. Average B-scores (n=3) for all four cell lines tested are provided.

Gene	Description	Clone reference	ACHN	Caki1	Caki2	KIJ265T
**ADM**	Adrenomedullin	NM_001124.1-441s1c1	−2.903	−3.823	−2.025	−2.025
		NM_001124.1-643s1c1	−3.001	−2.591	−1.677	−3.357
**ANGPTL4**	Angiopoietin like 4	NM_139314.1-1618s1c1	−5.296	−2.924	−2.001	−2.033
**BHLHB3 (BHLHE41)**	basic helix-loop-helix family, member e41	NM_030762.1-452s1c1	−1.825	ns	−2.134	−2.135
**BTK**	Bruton agammaglobulinemia tyrosine kinase	NM_000061.x-1873s1c1	−2.113	−1.549	ns	−2.4395
		NM_000061.x-1066s1c1	ns	−3.331	−1.772	ns
**CAMK1**	calcium/calmodulin-dependent protein kinase I	NM_020397.x-399s1c1	−2.248	−1.752	−1.646	−1.645
**CDH13**	cadherin 13, H-cadherin	NM_001257.2-1389s1c1	−2.914	−3.173	−1.848	ns
**CEP290**	centrosomal protein 290	NM_025114.1-1983s1c1	−2.408	−1.431	−3.755	−2.5605
**C20ORF100 (TOX2)**	TOX high mobility group box family member 2	NM_032883.1-1312s1c1	−2.688	−3.526	−2.119	−3.542
**EDNRA**	Endothelin receptor type A	NM_001957.1-1501s1c1	−2.536	−2.567	ns	ns
		NM_001957.1-1543s1c1	ns	ns	−2.523	−2.417
**EFCAB3**	EF-hand calcium binding domain 3	NM_173503.1-313s1c1	−1.384	−1.678	−1.862	−3.624
		NM_173503.1-535s1c1	−1.577	−3.152	−2.362	−2.267
**EGFR**	Epithelial growth factor receptor	NM_005228.3-396s1c1	−1.649	−2.03	−2.25	−1.3725
**ENPP3**	ectonucleotide pyrophosphatase, phosphodiesterase 3	NM_005021.2-1177s1c1	−1.613	−3.066	−2.129	−2.664
		NM_005021.2-83s1c1	−2.074	−3.485	ns	−2.8245
**FXYD5**	FXYD domain containing ion transport regulator 5 (dysadherin)	NM_014164.3-543s1c1	−2.671	−1.979	−3.656	−1.541
**IGFBP3**	insulin-like growth factor binding protein 3	NM_000598.4-711s1c1	−3.87	−2.924	ns	−2.892
		NM_000598.4-633s1c1	−1.975	−4.058	−2.094	−1.626
**KCNJ2**	Potassium inwardly-rectifying channel, subfamily J, member 2	NM_000891.2-1321s1c1	−2.506	−2.78	−1.027	−1.387
		NM_000891.2-1116s1c1	−1.836	−1.95	−2.25	ns
**KISS1R**	kisspeptin (metastin) receptor 1 (GPR54)	NM_032551.2-342	−6.549	−5.103	−4.533	−7.964
**KSR1**	kinase suppressor of ras 1	XM_290793.4-1852s1c1	−4.653	−3.977	−2.734	−2.433
**LAMA4**	laminin, alpha 4	NM_002290.2-1258s1c1	ns	−3.011	−2.147	−5.5775
**LOXL2**	lysyl oxidase-like 2	NM_002318.1-769s1c1	−1.157	−2.416	−2.369	−4.7325
		NM_002318.1-2416s1c1	−4.78	−1.12	−2.164	ns
**MYC**	v-myc myelocytomatosis viral oncogene homolog	NM_002467.2-1377s1c1	−3.371	−3.246	−2.473	−2.416
**NNMT**	Nicotinamide N-methyltransferase	NM_006169.1-711s1c1	−3.947	−2.136	−2.396	−2.168
		NM_006169.1-801s1c1	−2.255	−1.909	−1.493	ns
**NPTX2**	Neuronal pentraxin II	NM_002523.1-1623s1c1	−1.825	−1.999	−2.271	−1.438
		NM_002523.1-1316s1c1	−1.619	−2.92	−3.08	−1.504
**OLFML2A**	olfactomedin-like 2A	NM_182487.1-975s1c1	−2.711	ns	−3.566	−2.907
**PGBD5**	piggyBac transposable element derived 5	NM_024554.2-142s1c1	−6.624	−2.965	−2.87	−7.0545
**PLOD2**	procollagen-lysine, 2-oxoglutarate 5-dioxygenase 2	NM_000935.1-532s1c1	−1.649	−1.642	−2.14	−1.94
**RAPGEF5**	Rap guanine nucleotide exchange factor (GEF) 5	NM_012294.2-105s1c1	−3.625	−2.346	−1.932	−2.182
**SCD**	Stearoyl-CoA desaturase	NM_005063.3-1200s1c1	−2.216	−1.495	−2.192	−3.231
		NM_005063.3-780s1c1				
**SEMA6A**	sema domain, transmembrane domain (TM), and cytoplasmic domain, (semaphorin) 6A	NM_020796.1-1890s1c1	−2.348	−2.099	−1.606	−1.626
**SSPN**	sarcospan (Kras oncogene-associated gene)	NM_005086.3-352s1c1	−3.359	−2.424	−2.326	−2.325
**TCF8 (ZEB1)**	zinc finger E-box binding homeobox 1	NM_030751.2-70s1c1	−1.72	−2.388	−2.205	ns
**TMCC1**	transmembrane and coiled-coil domain family 1	NM_015008.1-1345s1c1	−2.527	−2.583	−2.864	−2.1195

Ingenuity® Systems was used to perform gene network clustering of the 31 hits in order to identify pathways that may be consistently altered in ccRCC. 26 of the 31 hits were clustered into one primary network that includes factors known to be involved in angiogenesis and inflammation, cell motility and EMT, and cancer and hereditary diseases (Figure 2B). Gene hits are highlighted in yellow. Of the 31 hits identified, we correlate overexpression of 14 for the first time with RCC: BHLHB3 (basic helix-loop-helix family, member e41), CAMK1 (calcium/calmodulin-dependent protein kinase I), CDH13 (cadherin 13), CEP290 (centrosomal protein 290kDa), EFCAB3 (EF-hand calcium binding domain 3), ENPP3 (ectonucleotide pyrophosphatase/phosphodiesterase 3), KCNJ2 (potassium inwardly-rectifying channel, subfamily J, member 2), KSR1 (kinase suppressor of ras 1), PGBD5 (piggyBac transposable element derived 5), PLOD2 (procollagen-lysine, 2-oxoglutarate 5-dioxygenase 2), SSPN (sarcospan), RAPGEF5 (rap guanine nucleotide exchange factor 5), TCF8 (zinc finger E-box binding homeobox 1, *ZEB1*) and TOX2 (TOX high mobility group box family member 2, *C20ORF100*).

RCC tumors exhibit dense networks of tumor vasculature [14], and are also frequently characterized by high levels of pro-inflammatory cytokines and eicosanoids [15]. Several genes comprising the network in Figure 2B have been previously implicated in the pathophysiology of RCC vasculogenesis and include ADM (adrenomedullin) [16], ANGPTL4 (angiopoietin-like 4) [17, 18], EGFR (epidermal growth factor receptor) [19, 20], EDNRA (endothelin receptor type A) [21], and LAMA4 (laminin, alpha 4) [22, 23]. Other members of this signaling network have been characterized to play a pro-inflammatory role in ccRCC. These include SCD1 (stearoyl-CoA desaturase 1) [24], BTK (bruton agammaglobulinemia tyrosine kinase) [25, 26], and FXYD5 (FXYD domain containing ion transport regulator 5, dysadherin) [27-29]. CDH13 appears to be frequently methylated in cancer, however its expression has been reported to be upregulated in tumor-associated vasculature, where it promotes endothelial cell proliferation and migration [30]. Its role in RCC remains to be defined. Other genes in this network have demonstrated pro-tumorigenic activity by mediating inflammation, cell transformation, proliferation, and metastasis in other cancers; however, their correlation with RCC pathophysiology has not been established. These include KSR1 [31-33] and PLOD2 [34, 35].

Expression of molecular factors involved in cell migration and invasion in RCC are of particular interest as they suggest significant dysregulation of cellular motility and epithelial to mesenchymal transition (EMT) in the pathophysiology of this cancer. The network in Figure 2B provides evidence of potential signaling pathways that may contribute to the invasive and metastatic nature of ccRCC. Genes that are transcriptionally upregulated and have been previously linked to cell migration in RCC include LOXL2 (lysyl oxidase-like 2) [36], NNMT (nicotinamide N-methyltransferase) [37], NPTX2 (neuronal pentraxin 2) (data unpublished), and OLFML2A (olfactomedin-like 2A) [38]. SEMA6A (semaphorin 6A) belongs to a large family of genes involved in the cytoskeletal remodeling and morphogenesis of nervous systems tissues [39]. While SEMA6A appears to be overexpressed in RCC, current literature suggests an anti-angiogenic role for this factor [40]. TMCC1 (transmembrane and coiled-coil domain family 1) was recently shown to be overexpressed at the transcript level in ccRCC [41], yet its functional role remains to be defined. While ENPP3 is involved in tumor cell migration [42, 43] and KCNJ2 is overexpressed in papillary thyroid carcinoma [44], the role of either of these factors is unknown in RCC.

Of the remaining hits shown in Figure 2B, MYC expression is frequently described as an oncogenic driver of proliferation, and has been previously characterized in RCC [10, 45]. IGFBP3 (insulin-like growth factor binding protein 3) overexpression has been proposed as a prognostic factor for RCC [46], and it is thought to potentiate oncogenic signaling through PI3K-AKT-mTOR through IGF1R activation [47]. KISS1R (KISS1 receptor) presents an interesting quandary. Activation of this G-coupled protein receptor by its canonical ligand, kisspeptin (metastin), has been shown to inhibit tumor cell migration and invasion in several cancers including RCC [48, 49]. Recently, a pro-invasive role has been proposed for KISS1R in breast cancer [50]. Data generated in this study additionally supports an invasive role for KISS1R in ccRCC. Calmodulin kinases have been implicated in oncogenesis [51, 52], though CAMK1 activity remains to be defined in RCC. SSPN expression has been studied in the context of muscle cell adhesion, strength, and regeneration [53]; nonetheless, a role for SSPN in cancer has yet to be defined. Finally, both RAPGEF5 and CEP290 have not previously been linked to malignancy.

Of the genes that were not included in the pathway illustrated in Figure 2B, BHLHB3 is implicated as a tumor suppressor in lung cancer [54], and TCF8 expression has been inversely correlated with E-cadherin expression in several cancers [55]. EFCAB3, PGBD5, and TOX2 remain to be defined in the context of tumorigenesis.

### Validation of tumor specific expression of a subset of hits identified in ccRCC

**Figure 3:**
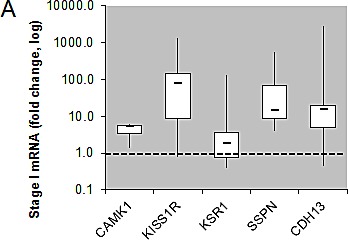

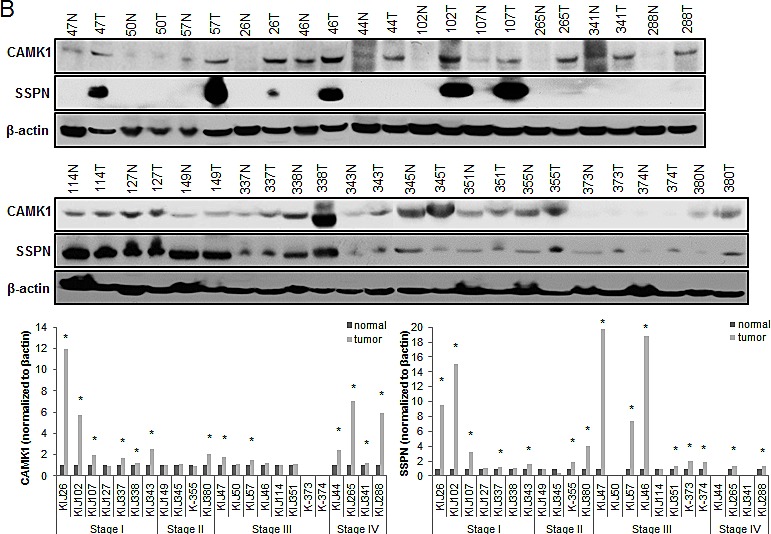

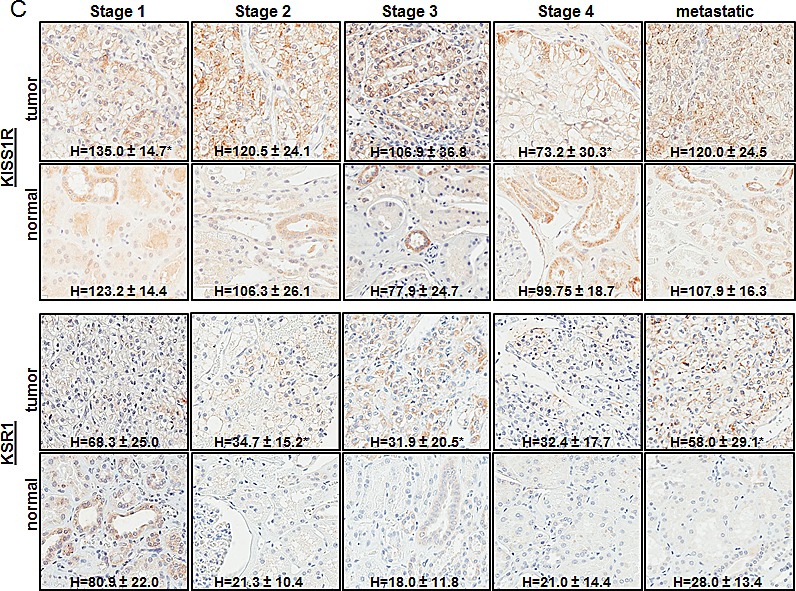

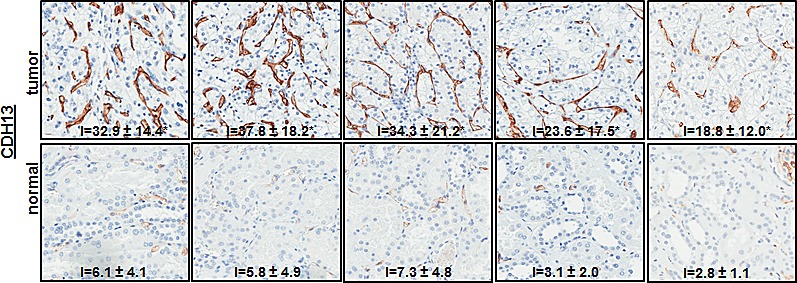
Functional Validation of tumor specific expression of a subset of hits identified in RCC (A) Box whisker plot of QPCR evaluating CAMK1, KISS1R, KSR1, SSPN, and CDH13 mRNA expression in stage I patient tumor and matched normal samples (n=8 for each normal and tumor samples). Results are shown as tumor fold change induction vs. normal samples, where the dashed line represents normal tissue expression. (B) Western blot for CAMK1 and SSPN in protein lysates derived from stage I-IV patient tumor and matched normal samples. Protein levels are quantitated against β-actin loading control, and tumor samples are normalized against matched normal samples which are set at 1. Samples are further sorted by stage, and tumors that demonstrate over a 20% increase in expression vs. matched normal are considered to be significantly overexpressed(*). (C) IHC of patient tissue microarrays for protein expression of KISS1R (normal n=40, 24, 25, 6, 9 and tumor n=36, 15, 23, 6, 20 for stages I, II, III, IV, and metastasis, respectively), KSR1 (normal n=53, 34, 34, 8, 8 and tumor n=42, 21, 23, 6, 24 for stages I, II, III, IV, and metastasis, respectively), and CDH13 (normal n=53, 29, 33, 8, 9 and tumor n=40, 19, 22, 8, 19 for stages I, II, III, IV, and metastasis, respectively). Representative tumor and normal images as well as mean H-scores ± standard deviation are shown for KISS1R and KSR1; mean I-scores ± standard deviation are shown for CDH13. Asterisk indicates values of statistically significant increases in tumor samples (where p≤0.05).

Of the 31 validated hits from the high-throughput lentiviral screen, we narrowed our focus to several genes whose roles in ccRCC malignancy is largely unknown. Real time quantitative PCR analysis (QPCR) confirmed over-expression of CAMK1, KISS1R, KSR1, SSPN, and CDH13 at the transcript level in patient derived stage I tumor tissue as compared to matched normal samples (Figure 3A), as expression of these hits appear to be transcriptionally upregulated in the gene array dataset in early stages of disease (Figure 1A). Immunohistochemistry (IHC) of ccRCC tumor tissue microarrays (TMA) for CAMK1 and SSPN demonstrated non-specific staining and could not be quantitatively analyzed. Therefore, protein tissue lysates were prepared from 23 normal and matched ccRCC samples derived from stage I-IV patients, and western blotting was performed. CAMK1 was overexpressed in 56% (13/23) and SSPN was overexpressed in 65% of the samples examined (15/23) (Figure 3B). IHC for KISS1R demonstrated a trend of protein overexpression in tumor samples corresponding to stages I, II, III, and metastasis, however only stage I and stage III results were considered to be statistically significant based upon H scores (p≤0.05) (Figure 3C). Interestingly, KISS1R appears to be predominantly localized to the plasma membrane in the tumor samples at all stages of disease, where staining in the corresponding normal samples was primarily cytoplasmic (Figure 3C, SF5A). KISS1R also demonstrates membranous and cytoplasmic staining in ccRCC tumor cell lines (SF5B). Since KISS1R is a G-coupled protein receptor, these results suggest that KISS1R may be functionally active in tumor samples as compared to normal. KSR1 protein is overexpressed in RCC in stage II-IV and metastatic samples, and demonstrates a cytoplasmic pattern of staining in both tumor and normal samples (Figure 3C). CDH13 expression appears to be significantly upregulated in RCC tumor vasculature at all stages of disease as compared to normal samples (Figure 3C). In order to further evaluate CDH13 localization, serial sections of both tumor and matched normal tissue were stained for CD31, a marker of endothelial as well as other blood cells, and CDH13. CDH13 staining was analogous to CD31 staining, suggestive that CDH13 expression is specific to the vasculature constituents (SF5C). This pattern of expression corroborates current literature, suggesting a possible role for CDH13 expression in tumor-associated vasculogenesis. These results confirm tumor-specific overexpression of CAMK1, SSPN, KISS1R, KSR1, and CDH13 at the transcript and protein level.

### Functional validation of novel pro-invasive hits in RCC cells

**Figure 4:**
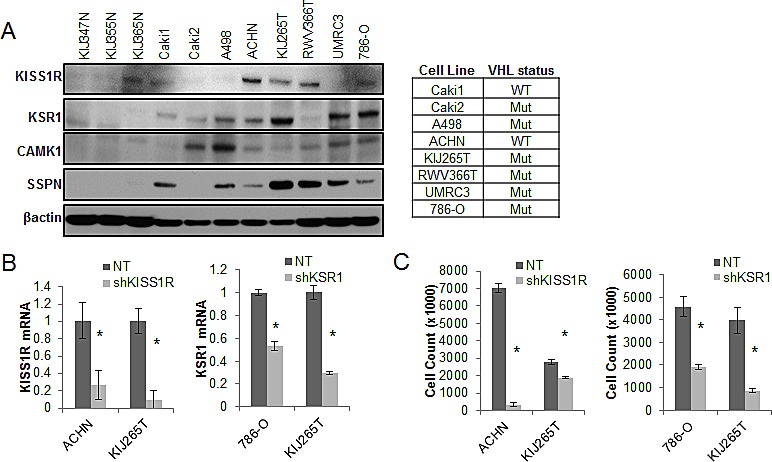

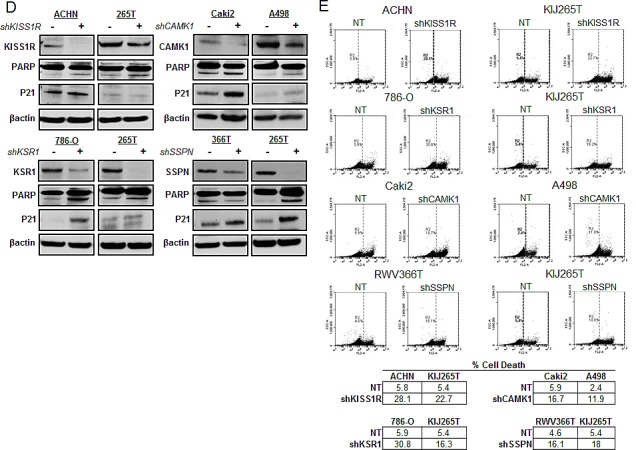
Decreased expression of KISS1R, KSR1, CAMK1, and SSPN leads to tumor cell death and/or senescence (A) Protein expression analysis of KISS1R, KSR1, CAMK1, and SSPN in NRE and ccRCC cell lines via western blot. *VHL* mutation status is provided. (B) QPCR for mRNA transcript expression in NT and hit knockdown cells are presented as fold change relative to NT control, where NT values are set at 1. (C) Proliferation of NT vs. hit targeted shRNA cell lines. (D) Western blot for protein expression in NT control and shRNA targeted cells for PARP, and P21. β-actin was used as a loading control. (E) Flow cytometry analysis of propidium iodide stained NT and target knockdown cells evaluating cell death. Increases of 5% or greater versus NT control cells were considered to be significant.

We further explored the contribution of *KISS1R, KSR1, CAMK1* and *SSPN* expression at the functional level. Normal renal epithelial (NRE) and RCC cell lines were analyzed via western blot in order to validate tumor-specific expression of target hits as well as establish appropriate working models (Figure 4A). The *VHL* status for each RCC cell line is provided (Figure 4A). Two representative cell lines that demonstrate high levels of protein expression for each hit were chosen to further investigate functionality in the context of RCC. Cells were infected with shRNA constructs targeting each gene, and the resulting decrease in mRNA expression was evaluated by QPCR (Figure 4B), and decreased protein expression was evaluated by western blot (Figure 4D). Cell proliferation in response to decreased hit expression was assessed (Figure 4C), and significant decreases were observed in all cell lines for each target. We performed western blot analysis to evaluate induction of apoptosis via PARP cleavage as well as inhibition of cell cycle progression via p21 upregulation. Significant increases in PARP cleavage were observed in all cell lines as compared to nontarget (NT) controls for each hit, except for RWV366T shSSPN cells (Figure 4D). An increase in p21 expression was observed in RWV366T cells, as well as in Caki2 and A498 shCAMK1 cells, 786-O shKSR1 cells, and KIJ265T shSSPN cells as compared to NT control cells (Figure 4D). Induction of cell death was also evaluated via flow cytometry of propidium iodide stained NT versus target knockdown cells. Results demonstrate a significant increase in cell death in all target knockdown cells evaluated (Figure 4E), further supporting the requirement for each target gene expression in tumor cell viability.

**Figure 5:**
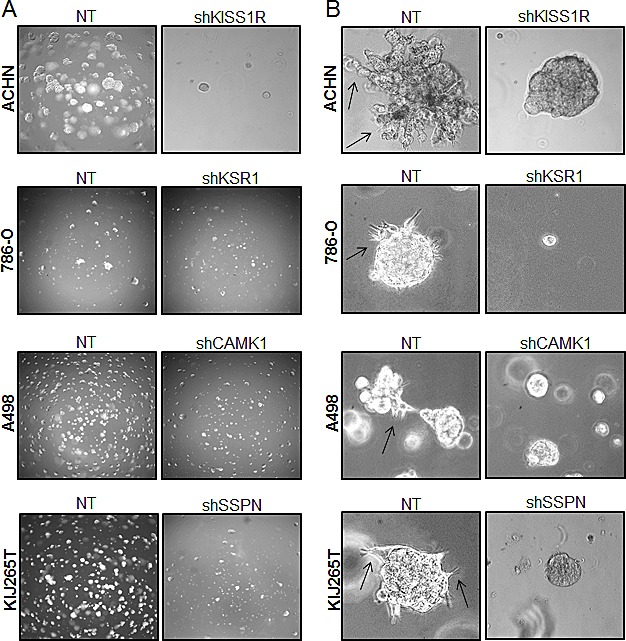

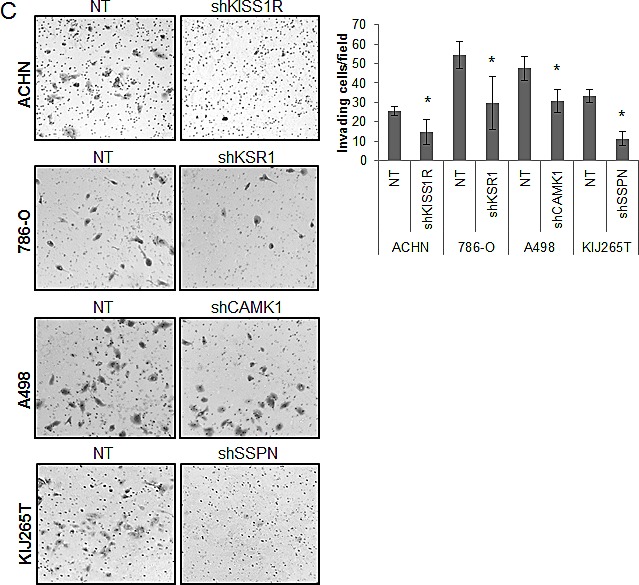

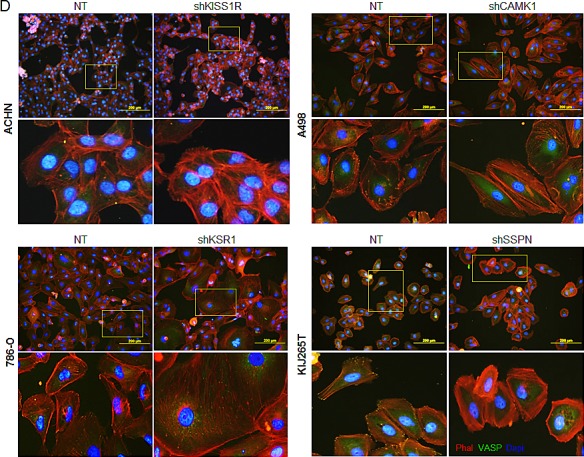
Decreased KISS1R, KSR1, CAMK1, and SSPN expression results in loss of invasive phenotype in ccRCC cells (A) Images represent 10x magnification of NT and specified hit knockdown cells plated in a 3D matrix after 10 days of growth. (B) 40x manual magnification of representative colonies of NT and target hit knockdown cells grown in 3D cell culture assays at day 10. Stellar outgrowth is seen in NT populations and is indicated by black arrows. (C) Invasion assays of NT and target hit knockdown cells. Representative 10x images of transwell inserts from invasion chambers are shown. Invasion is quantitated as number of invading cells per visual field (n=3). (D) Immunofluorescence for phalloidin, VASP, and dapi (nuclear stain) in NT and hit target knockdown cells. Top panels for each cell line are 20x magnification. Highlighted areas magnified manually, and are shown directly below panel of origin.

As KISS1R, KSR1, CAMK1, and SSPN have been either previously implicated in tumor cell migration or are known to participate in extracellular matrix adhesion, we wanted to explore whether any of these factors mediate RCC cell invasiveness. Three-dimensional cell culture was performed in order to evaluate the ability of NT and target knockdown cells to form multicellular spheroids, as well as their ability to invade into the surrounding substrate. After 10 days, a marked reduction in the size of colonies was observed in all targeted mRNA silenced cells (Figure 5A, B). Additionally, ACHN shKISS1R and KIJ265T SSPN cells demonstrated a significant decrease in the number of colonies observed, indicative of decreased anchorage-independent growth as compared to NT control cells (Figure 5A). Closer inspection of colony morphology revealed stellar outgrowth in the majority of all NT control colonies, indicative of an invasive phenotype (Figure 5B). This phenotype was severely compromised in targeted mRNA silenced cells which not only formed smaller aggregates, but appeared spherical and lacking stellate outgrowth (Figure 5B). A significant decrease in tumor cell invasion in all target hit knockdown cells using standard matrigel chemoinvasion assays further illustrated a loss of invasive capacity as compared to NT control cells (Figure 5C). In order to evaluate morphological changes at the cellular level, NT and target cells were stained using Phalloidin- a marker of filamentous actin, allowing for the visualization of fluorescent intensity and arrangement of actin filaments. Additionally, cells were co-stained for VASP- an adapter protein thought to regulate actin polymerization and cell motility [56]. 20x images as well as a manual zoom of boxed-in regions within the 20x images are provided to better visualize morphological changes. Very little VASP staining was present in ACHN cells, however a significant increase in actin stress fiber formation was observed in shKISS1R cells as compared to NT control cells (Figure 5D). 786-O shKSR1 cells displayed an enlarged cell morphology, indicative of senescence (Figure 5D). A498 shCAMK1 and KIJ265T shSSPN cells not only displayed increased actin stress fiber formation, but a significant decrease in VASP staining was observed as compared to respective NT control cells, where they demonstrated a punctate expression at the cellular periphery-a staining pattern that is supportive of cell migration in control cells.

## DISCUSSION

In this study we utilized a high-throughput gene array of patient ccRCC tissues and matched normal samples followed by a high-throughput shRNA screen in an effort to not only identify genes transcripts that are overexpressed in tumor samples, but also to ascertain those that promote ccRCC cell proliferation. Our findings reveal tumor-specific upregulation of factors that not only influence tumor cell growth, but also angiogenesis, inflammation, and cell migration. Moreover, we implicate several of these gene hits not only in ccRCC for the first time (BHLHB3, CAMK1, CDH13, ENPP3, KCNJ2, KSR1, PLOD2, and TCF8), but also in a cancer model for the first time (CEP290, EFCAB3, PGBD5, RAPGEF5, SSPN, and TOX2). Additionally, we functionally validate the contribution of a specific subset of hits not only to tumor cell viability, but to tumor cell migration *in vitro*. These include KISS1R, KSR1, CAMK1, and SSPN.

Our results also confirm that overexpression of CDH13 protein appears to be exclusive to tumor vasculature. Currently, ccRCC is well characterized as being a highly vascularized tumor and therefore inhibitors of angiogenesis present as an attractive target for therapy in patients [57]. Several treatment regimens that specifically target pro-angiogenic factors (such as VEGF, VEGFR and PDGFR) have been approved by the FDA for the treatment of advanced ccRCC. These currently provide some clinical benefit for patients and include pazopanib, sorafenib, sunitinib, bevacizumab, and axitinib. CDH13 presents as a potential novel anti-angiogenic target in ccRCC, and further investigation of its specific role in tumor vasculogenesis is warranted.

Previous studies evaluating KISS1R expression and its role in tumorigenesis in ccRCC has emphasized a tumor-suppressive role for this receptor in conjunction with its ligand, kisspeptin, where it inhibits tumor cell invasion [48, 49]. Our findings contradict this data and provide evidence that KISS1R overexpression may support a pro-invasive phenotype in ccRCC, as mRNA silencing of this receptor inhibits ccRCC cell invasion *in vitro*. This finding is suggestive of a more complicated role for KISS1R in ccRCC whereby it may facilitate tumor cell invasion via a non-canonical mechanism, possibly in the absence of its ligand. Further studies are necessary to elucidate its regulation. In addition to KISS1R, we identify a pro-invasive role for KSR1, CAMK1, and SSPN in the context of ccRCC. Given that ccRCC is a highly metastatic tumor as evidenced by the frequency of disease recurrence, these findings may shed light on critical signaling pathways that drive tumor cell migration, even in early stage disease. These targets may themselves present as possible therapeutic candidates or prognostic biomarkers for disease aggressiveness given their overexpression at the protein level in ccRCC tissues.

In summary, patients with advanced ccRCC have limited therapeutic options due to the high degree of tumor heterogeneity, drug resistance, and lack of molecular targets that are consistently deregulated in a large proportion of cases. The findings of this study not only corroborates the work of other groups investigating ccRCC, but also illuminates the contribution of a cohort of genes that are less well understood in the context of this disease. In particular, our results reveal altered expression of several genes thought to be involved in cell adhesion and migration in other cancers and diseases, and further implicate their involvement in ccRCC cell migration. As ccRCC is highly metastatic, these findings may be critical for understanding the mechanisms that drive this invasive phenotype. Moreover, our findings highlight additional novel candidate factors that may serve as prognostic biomarkers or targets for therapeutic intervention for ccRCC patients given their requirement for tumor cell proliferation.

## MATERIALS AND METHODS

### Cell lines

ACHN, A498, Caki1, and Caki2 ccRCC cell lines were purchased from American Type Culture Collection (Manassas, VA). KIJ265T and RWV366T were developed in the Copland Laboratory as previously described [24, 58]. 786-O and UMRC3 were a kind gift from Dr. B. H. Grossman [59]. VHL mutational and deletional status were examined via DNA sequencing and multiplex ligation dependent probe amplification, respectively. Cells were maintained in DMEM (Cellgro) supplemented with 5% FBS (Hyclone) and 1% penicillin-streptomycin-amphotericin B (Cellgro) at 37°C with 5% CO_2_.

### High-Throughput DNA Microarray

Purified RNA samples were sent to the Mayo Clinic Advanced Genomic Technology Center Gene Expression Core where gene array expression analysis was performed using Affymetrix Human Genome U133 Plus 2.0 Array chip. Samples were derived from matched normal and diseased ccRCC patient tissue across all stages of disease (n= I-24, II-19, III-14, IV-15). Gene expression data was deposited at the Gene Expression Omnibus Database (Accession #GSE-53757). Details of the data processing and methodology are previously described in [60]. Use of all tissues in the course of the study was approved by the Mayo Institutional Review Board. Heatmaps were generated with Genespring GX 7.3.1 (Agilent Technologies) using Affymetrix default analysis settings and standard Genespring normalizations (normalized value = Raw signal value/Control Value where the Control value is a product of the 50% of the chip and Median of the signal value for that gene). Meta-analysis of publicly available gene array datasets was performed as previously described using NextBio data mining platform [61].

### High-Throughput shRNA Array

The top 195 genes overexpressed in RCC samples along with control genes were used to design a high-throughput shRNA screen. MISSION ® shRNA particles were prepared and plated individually by Sigma-Aldrich in Corning black, clear-bottom 96-well plates. ACHN, Caki1, Caki2, and KIJ265T cells were plated at 5000 cells per well (for a 200 MOI) in phenol red-free DMEM with 10% FBS, 1% PSA and 1X glutamax. Triplicates were on 3 separate plates. After 72 hours, plates were washed with PBS, and stored at -80ΰC prior to analysis using CyQuant ® Proliferation Analysis Kit (Invitrogen) as per manufacturers' protocol for relative fluorescence units.

### Statistical Analysis

The raw relative fluorescence unit values for each plate were used for analysis using HTS Corrector software for median [62] polishing and B score calculations (Z score analog). B scores were chosen over Z scores based upon uncommon variances due to column and row bias to rule out outliers as demonstrated in Figure S2 [11]. For screening the hits at 95% probability, *P*=0.05 and Z= -1.645. Experimental error is presented as fold change or standard deviation where specified. Group comparisons were performed using two-tailed paired student's *t*-test, where p≤0.05 were considered to be statistically significant (normal vs. tumor and NT vs. shRNA) unless specified otherwise.

### Gene Network Clustering

Ingenuity® Systems was used to cluster the 31 over-expressed genes validated by the high-throughput shRNA screen into functional groups.

### Lentiviral Infections

MISSION shRNA pLKO.1 constructs (Sigma-Aldrich) were used to make self-inactivating shRNA lentiviruses for CAMK1 (NM_020397.x_399s1c1), KISS1R (NM_032551.2-342), KSR1 (XM_290793.4-1852s1c1), SSPN (NM_005086.3-352s1c1) and a non-target (NT) random scrambled sequence control (SHC002). Lentivirus was prepared as previously described [24]. Cells were incubated overnight with lentivirus particles. Cells were washed 3x with PBS, and were allowed to recover for 24 hours in regular growth media. Puromycin (Life Technologies) selection was added to cells for a minimum of 5 days prior to experimentation.

### RNA isolation and Realtime QPCR Analysis

RNA from human tissue was prepared using using TRIzol® (Invitrogen) along with sonication on ice and then extracted using a Phenol (Ambion): Chloroform (Boehringer Mannheim Inc.) mix prior to purification using the RNAqueous Midi Kit (Ambion). cDNA was prepared from purified RNA samples using using High Capacity cDNA Reverse Transcriptase Kit (Applied Biosystems) per manufacturer's instruction. TaqMan® Fast Universal PCR Master Mix (Applied Biosystems) and TaqMan® FAM™ dye-labeled probes including POLR2A (Hs00172187_m1), CAMK1 (Hs00220668_m1), CDH13 (Hs01004530_m1), KISS1R (Hs00261399_m1), KSR1 (Hs01075790_m1), and SSPN (Hs01025520_m1) were combined with prepared cDNA samples to analyze relative mRNA expression via quantitative real time PCR (QPCR). POLR2A expression was used as normalization control. Fold change values were compared between normal and tumor as well as non-target scrambled lentiviral and target lentiviral infected using the ΔΔCt method [63].

### Proliferation Assays

Cells were infected with either NT control or target lentivirus as previously described. Cells were plated at 2x10^4^ cells/well in 12-well plates (Midwest Scientific) in triplicate. After 5 days, cell number was established using a Coulter Particle Counter (Beckman). Puromycin selection was maintained throughout the course of the assay.

### Immunohistochemistry and immunocytochemistry Analysis

Formalin-fixed paraffin-embedded tissue microarrays (TMA) of patient ccRCC tumor and matched normal tissues were prepared. Samples were blocked with Diluent that contained Background Reducing Components (Dakocytomation, Denmark) for 30 minutes and then probed for KISS1R (Alomone Labs), KSR1 (Santa Cruz Biotechnologies), CDH13 (Sigma), and CD31 (R&D Systems). Tissue stain scoring was performed as previously described [60]. 20x images were obtained using Scanscope XT and Imagescope software (Aperio Technologies). Scoring methods were performed as previously described [24]. Cells were plated in 4-chamber slides (Thermo Scientific), were fixed with 4% paraformaldehyde (Sigma), permeabilized with 0.1% Triton X-100 (Sigma), and blocked with Diluent (Dakocytomation) for 1H. Cells were first incubated with primary antibody -VASP (Cell Signaling), KISS1R (Alomone Labs), followed by species specific secondary. Phalloidin (Life Technologies) stain was applied following antibody application. VECTASHIELD mounting media (Vector Labs) containing dapi was used. Negative sections were prepared by incubating the slides in the absence of the primary antibody.

### Western Blot Analysis

Tissue protein extracts were prepared from frozen samples using 1% SDS (Sigma) in 50mM pH 8.0 Tris buffer (Sigma) containing protease inhibitor cocktail (Roche) and phosphatase inhibitor (Pierce). Samples were sonicated on ice. Bicinchoninic acid assay (Pierce) was used to quantify protein concentrations. NuPAGE® LDS sample loading buffer plus sample reducing agent (Invitrogen) were added to lysates. Samples were loaded in Novex 4-12% Bis-Tris gels in MES buffer (Invitrogen) for electrophoresis separation, and transferred to 0.2 μM Immobilon Psq membranes (Millipore) for western blot analysis. 5% milk in Tris Buffered Saline plus 0.1% Tween-20 (TBS-T) (Fischer Scientific) was used as a blocking buffer as well as antibody diluent. Primary antibodies to detect KISS1R (Alomone Labs), KSR1 (Santa Cruz Biotechnologies), CAMK1 (Abnova), SSPN (Santa Cruz Biotechnologies), and β-actin (Sigma-Aldrich) were used. All secondary species-specific horseradish peroxidase-labeled antibodies were purchased from Jackson Immunoresearch and Supersignal chemiluminescent kit (Pierce) was used to perform detection.

### Cell Death Analysis via Flow Cytometry

ccRCC cells were infected with either NT control or target lentivirus as previously described. After puromycin selection, both adhered and floating cells were collected using Accutase (Innovative Cell Technologies, Inc.), washed with PBS, and were suspended in 1x cold binding buffer (BD Pharmingen) at 1x10^6^ cells/mL. Cells were stained with Propidium Iodide (BD Pharmingen), and cell death analysis was performed using an Accuri C6 flow cytometer (Accuri). Unstained NT cells were used to set population parameters.

### 3D Cell Culture and Invasion Assays

3D cell culture assays were performed as previously described [64]. Media was changed every 2 days, and puromycin selection was maintained. Photos were taken on day 10 at 10x magnification for an overview of colony size and density, and 40x magnification images were taken to evaluate colony structure and morphology. For the invasion assays, cells were starved overnight in 0.2% FBS DMEM. BD Biocoat Matrigel Invasion Chambers (8μm pore) (BD Biosciences) were prepared per manufacturer's protocol. 5,000 cells were plated (in triplicate) with 0.25% BSA in the upper chamber and 5% FBS was the attractant in the lower chamber. Puromycin selection was maintained. Transwell inserts were fixed in 100% methanol and stained with 0.2% crystal violet/2% ethanol after a 20 hour time period. Invasion was quantitated as number of invading cells per visual field, where 3 visual fields were analyzed per transwell insert. Assay was performed in triplicate per group. 10x images were obtained using an Olympus microscope (Olympus IX71).

## SUPPLEMENTARY MATERIAL TABLES AND FIGURES


