# Supplementary material for: Functional genomics identifies novel genes essential for clear cell renal cell carcinoma tumor cell proliferation and migration
Source: Oncotarget. 2014 Jun 12;5(14):5320–34. doi: 10.18632/oncotarget.2097 (PMC4170622; doi:10.18632/oncotarget.2097)
Supplement: Supplementary file 1 [file oncotarget-05-5320-s001.pdf]

Functional genomics identifies novel genes essential for clear cell renal cell carcinoma tumor cell proliferation and migration

Supplementary Material

SF1. ***Summary of the 195 genes identified in the comparative marker selection.*** **Genes are listed in alphabetical order.**

Genes examined				
ABCA1	***COL5A3***	***GMFG***	***NAV1***	***SEMA3F***
ABCG1	***COL6A2***	***GPR4***	***NCF4***	***SEMA6A***
ADM	***COP1***	***GRIA4***	***NDRG1***	***SERINC3***
ALDOC	***CP***	***HAPLN1***	***NDUFA4L2***	***SERTAD2***
ALOX5	***CPE***	***HEY1***	***NETO2***	***SETD2***
ANGPT2	***CSDA***	***HIF1A***	***NKG7***	***SHMT2***
ANGPTL2	***CSPG4***	***HIG2***	***NNMT***	***SIGLEC1***
ANGPTL4	***CTHRC1***	***HIPK2***	***NOL3***	***SLA***
APOC1	***CTSZ***	***HK2***	***NPIP***	***SLAMF8***
APOLD1	***CXCR4***	***HMCN1***	***NPTX2***	***SLC15A4***
ARHGAP25	***DARS***	***HPS3***	***NR3C1***	***SLC16A1***
ARL4A	***DDB2***	***HSD3B7***	***NUSAP1***	***SLC16A3***
BHLHB3	***DDIT4***	***IFI16***	***OLFML2A***	***SLC2A3***
BICD1	***DERL1***	***IGFBP3***	***P4HA1***	***SLC6A3***
BIRC3	***DIRAS2***	***IKIP***	***PCDH17***	***SLCO2B1***
BTK	***DNM1***	***IL12RB1***	***PDK1***	***SOCS3***
C10orf10	***EDNRA***	***INHBB***	***PECAM1***	***SPAG4***
C1GALT1	***EFCAB3***	***IRX3***	***PFKFB4***	***SPIRE1***
C1QA	***EGFR***	***ITGA5***	***PFKP***	***SSPN***
C1QB	***EGLN3***	***KCNE4***	***PGBD5***	***ST8SIA4***
C1QC	***EHD2***	***KCNJ2***	***PGF***	***STAMBPL1***

C20orf100	***ELOVL2***	***KCNK3***	***PHKA2***	***STC2***
CA9	***ENPP3***	***KCNMA1***	***PLAG1***	***TBL1XR1***
CAMK1D	***ENTPD1***	***KISS1R***	***PLOD2***	***TCF4***
CAV1	***EPAS1***	***KLF6***	***PML***	***TCF8***
CAV2	***ERGIC1***	***KSR1***	***PPP1R3C***	***TGFA***
CCND1	***FABP5***	***LAMA4***	***PRKCDBP***	***TGFBI***
CDCA2	***FABP6***	***LAMP3***	***PTPRC***	***TMCC1***
CDH13	***FABP7***	***LAPTM5***	***RAPGEF5***	***TMEM45A***
CDH2	***FCGR1A***	***LGI4***	***RGS1***	***TNFAIP6***
CENTA2	***FCGR2A***	***LOXL2***	***RGS5***	***TNFSF13B***
CEP290	***FGD2***	***LPCAT1***	***RNASE6***	***TPSAB1***
CEP350	***FLT1***	***LRRK1***	***RNASET2***	***TRAF3IP2***
CHES1 (FOXn3)	***FMNL2***	***MAP3K6***	***RNF149***	***TREM2***
CHST7	***FPRL2***	***MEF2C***	***RUNX1***	***TRIB3***
CMKOR1	***FXYD5***	***MET***	***RUNX2***	***TYROBP***
CMTM3	***GAS2L3***	***MS4A6A***	***RUNX3***	***VEGF***
COL1A1	***GIT2***	***MS4A7***	***SCARB1***	***ZNF395***
COL1A2	***GJA7***	***MYC***	***SCD***	***ZNF532***

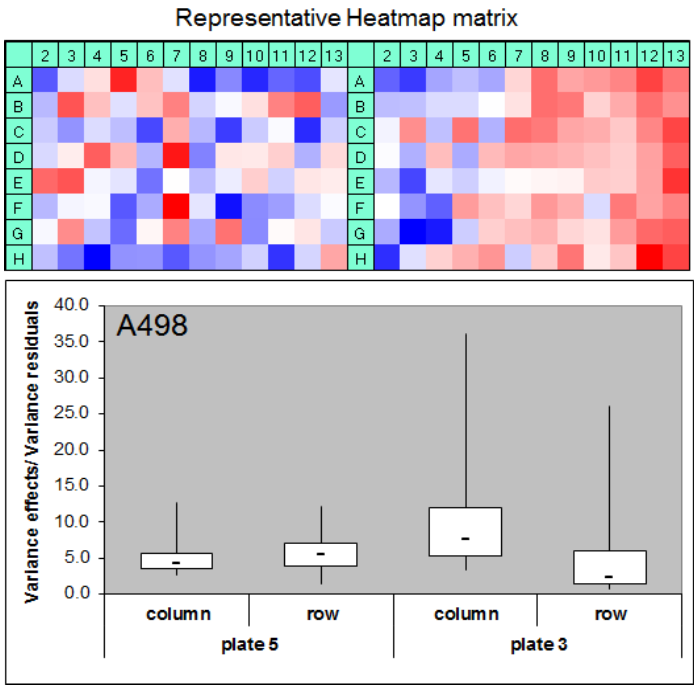

SF2. *Representative heatmap matrix for B-score justification.* Plate 5 represents an ideal normal distribution with common variance while plate 3 demonstrates uncommon variance and unevenly distributed data in need of median polishing for a B score, which is an analog of Z score.

SF3 and 4. ***Gene array expression profile of ccRCC vs. normal renal tissue.*** Fold change expression of tumor as compared to normal values are provided. Fold change inductions of 1.5 or greater where p≤0.05 were considered to be significant.

ccRCC vs. normal kidney (n=8)			
**Dataset**	**Gene**	**Fold Change**	**P value**
Ooi, 2011	ADM	5.18	4.80E-06
	ANGPTL4	16	5.80E-12
	BHLHB3 (BHLHE41)	16.9	1.10E-07
	BTK	1.50	4.80E-05
	CAMK1D	1.43	6.20E-03
	CDH13	3.61	9.60E-06
	CEP290	NA	
	C20ORF100 (TOX2)	NA	
	EDNRA	2.71	6.00E-04
	EFCAB3	3.1	1.70E-03
	EGFR	4.48	5.00E-04
	ENPP3	27.5	3.90E-08
	FXYD5	2.34	5.70E-06
	IGFBP3	6.6	8.40E-08
	KCNJ2	3.26	6.00E-04
	KISS1R	41.3	1.00E-04
	KSR1	2.87	1.30E-05
	LAMA4	5.43	1.70E-05
	LOXL2	5.35	2.90E-05
	MYC	3.17	1.00E-04
	NNMT	11.3	9.20E-07
	NPTX2	41.2	8.50E-05
	OLFML2A	3.3	4.60E-05
	PGBD5	2.6	3.30E-03
	PLOD2	2.84	5.80E-05
	RAPGEF5	2.59	1.00E-05
	SCD	7.51	5.10E-05
	SEMA6A	1.64	1.62E-02
	SSPN	2.71	1.00E-04
	TCF8 (ZEB1)	2.81	9.90E-05
	TMCC1	5.56	1.40E-07

ccRCC vs. normal kidney (n=10)			
**Dataset**	**Gene**	**Fold Change**	**P value**
Kort 2008	ADM	3.8	2.00E-06
	ANGPTL4	17	1.00E-14
	BHLHB3 (BHLHE41)	17.4	1.70E-13
	BTK	1.38	2.00E-04
	CAMK1D	1.53	4.00E-04
	CDH13	4.2	3.50E-06
	CEP290	1.26	1.01E-02
	C20ORF100 (TOX2)	2.13	1.80E-03
	EDNRA	3.43	2.90E-06
	EFCAB3	2.7	2.00E-04
	EGFR	3.94	2.00E-04
	ENPP3	26.6	3.80E-11
	FXYD5	2.21	1.00E-07
	IGFBP3	7.13	8.90E-12
	KCNJ2	3.6	6.10E-05
	KISS1R	35.7	2.30E-06
	KSR1	3.3	2.20E-07
	LAMA4	7.12	6.20E-08
	LOXL2	5.09	2.00E-07
	MYC	3.38	1.80E-06
	NNMT	11	1.20E-08
	NPTX2	33.6	1.10E-05
	OLFML2A	3.97	7.40E-07
	PGBD5	2.44	2.70E-05
	PLOD2	3.76	1.90E-07
	RAPGEF5	2.52	1.60E-08
	SCD	8.18	2.20E-11
	SEMA6A	2.86	1.00E-04
	SSPN	2.51	5.30E-07
	TCF8 (ZEB1)	3.14	4.20E-08
	TMCC1	5.87	2.40E-09

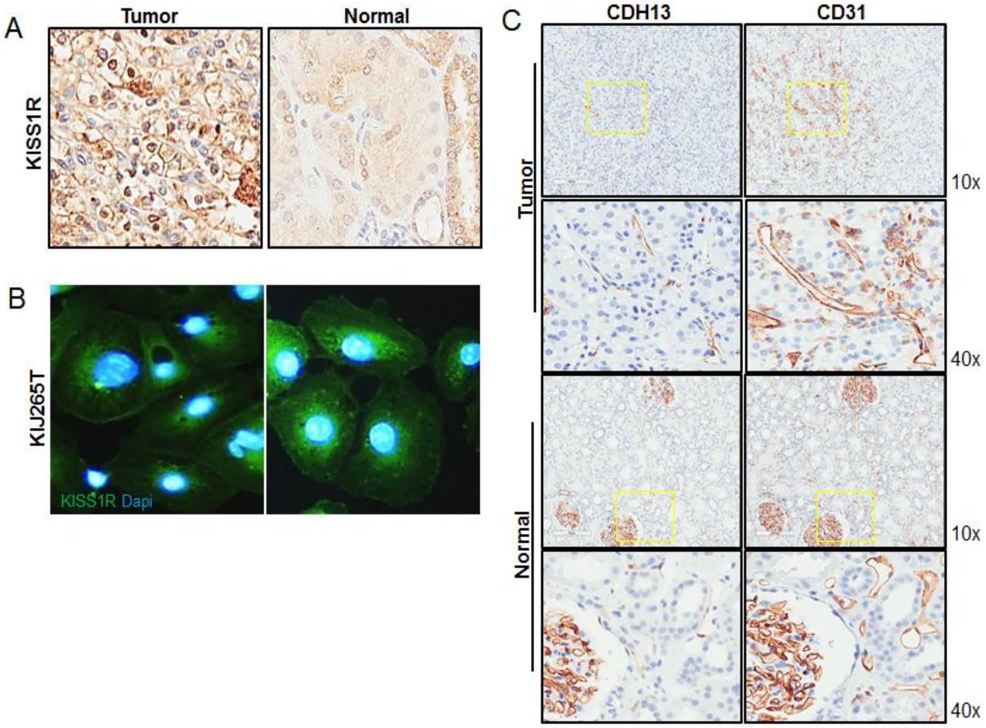

SF5. ***KISS1R and CDH13 protein expression in patient renal tissues.*** (**A**) Comparison of KISS1R localization in ccRCC versus matched normal. Representative images are a 60x manual magnification. (**B**) Immunofluorescence of KIJ265T ccRCC cell for KISS1R. Image shown is a 100x manual magnification. (**C**) Evaluation of CDH13 localization in patient tissue. Adjacent ccRCC and normal matched tissue samples were stained with CDH13 and CD31. 10x images are shown, and boxed in areas represent a 40x manual zoom.
